# The transcription factor ATF3 acts as an oncogene in mouse mammary tumorigenesis

**DOI:** 10.1186/1471-2407-8-268

**Published:** 2008-09-22

**Authors:** Aijin Wang, Stacey Arantes, Leqin Yan, Kaoru Kiguchi, Mark J McArthur, Aysegul Sahin, Howard D Thames, C Marcelo Aldaz, Michael C MacLeod

**Affiliations:** 1 Department of Carcinogenesis, The University of Texas M.D. Anderson Cancer Center, Smithville, TX, USA; 2 Department of Veterinary Resources, The University of Texas M.D. Anderson Cancer Center, Bastrop, TX, USA; 3 Department of Pathology, The University of Texas M.D. Anderson Cancer Center, Houston, TX, USA; 4 Department of Biostatistics, The University of Texas M.D. Anderson Cancer Center, Houston, TX, USA

## Abstract

**Background:**

Overexpression of the bZip transcription factor, ATF3, in basal epithelial cells of transgenic mice under the control of the bovine cytokeratin-5 (CK5) promoter has previously been shown to induce epidermal hyperplasia, hair follicle anomalies and neoplastic lesions of the oral mucosa including squamous cell carcinomas. CK5 is known to be expressed in myoepithelial cells of the mammary gland, suggesting the possibility that transgenic BK5.ATF3 mice may exhibit mammary gland phenotypes.

**Methods:**

Mammary glands from nulliparous mice in our BK5.ATF3 colony, both non-transgenic and transgenic, were examined for anomalies by histopathology and immunohistochemistry. Nulliparous and biparous female mice were observed for possible mammary tumor development, and suspicious masses were analyzed by histopathology and immunohistochemistry. Human breast tumor samples, as well as normal breast tissue, were similarly analyzed for ATF3 expression.

**Results:**

Transgenic BK5.ATF3 mice expressed nuclear ATF3 in the basal layer of the mammary ductal epithelium, and often developed squamous metaplastic lesions in one or more mammary glands by 25 weeks of age. No progression to malignancy was seen in nulliparous BK5.ATF3 or non-transgenic mice held for 16 months. However, biparous BK5.ATF3 mice developed mammary carcinomas with squamous metaplasia between 6 months and one year of age, reaching an incidence of 67%. Cytokeratin expression in the tumors was profoundly disturbed, including expression of CK5 and CK8 (characteristic of basal and luminal cells, respectively) throughout the epithelial component of the tumors, CK6 (potentially a stem cell marker), CK10 (a marker of interfollicular epidermal differentiation), and mIRSa2 and mIRSa3.1 (markers of the inner root sheath of hair follicles). Immunohistochemical studies indicated that a subset of human breast tumors exhibit high levels of nuclear ATF3 expression.

**Conclusion:**

Overexpression of ATF3 in CK5-expressing cells of the murine mammary gland results in the development of squamous metaplastic lesions in nulliparous females, and in mammary tumors in biparous mice, suggesting that ATF3 acts as a mammary oncogene. A subset of human breast tumors expresses high levels of ATF3, suggesting that ATF3 may play an oncogenic role in human breast tumorigenesis, and therefore may be useful as either a biomarker or therapeutic target.

## Background

Most human ductal breast carcinomas express cytokeratin (CK) markers characteristic of the luminal epithelial cell compartment, namely CK8 and CK18 [1]. In the past, this led to an overall interest among cancer biologists in cell compartments containing luminal progenitor cells as the possible target populations for carcinogenesis leading to invasive ductal carcinoma (IDC). This is reflected in the extensive development of transgenic mouse models of breast cancer based on promoters thought to be active in such cells, namely the MMTV promoter and pregnancy/lactation specific promoters [2]. However, recently several large surveys have revealed that a significant fraction of invasive ductal carcinomas, 25–30%, express CK5 and/or CK14, cytokeratins that are characteristic of myoepithelial cells [1,3], resulting in renewed interest in the myoepithelial cell lineage as the target population for some cancers [4]. The subset of breast cancers that are CK5/14-positive is enriched for the so-called basal-like tumors, identified on the basis of large scale gene expression patterns [5-9]. This subset is of particular interest because these tumors tend to be negative for estrogen receptor alpha (ERα), lymph node positive and have a generally poor prognosis [7,8].

The exact location and properties of undifferentiated, progenitor cells that give rise to luminal and myoepithelial cells in the adult breast is not well defined [4,10,11], and hence the hierarchical relationships between progenitor populations that may be targets for cancer induction are still speculative. One theory suggests that mammary stem cells give rise to separate populations of undifferentiated progenitor cells for the two major pathways, and that these luminal or myoepithelial progenitor cells may represent the targets for carcinogenesis [12]. However, several findings argue against this model.

First, the vast majority of the CK5/14-positive IDCs also express CK8/18, and very few tumors (< 1%) express solely myoepithelial markers [1]. Secondly, genetic studies of IDC indicate that most occurrences of loss of heterozygosity include both luminal and myoepithelial cells [13], implying that the original genetic event occurred in a precursor population that had the capability to give rise to both compartments. These findings suggest that either the predominant target population for induction of IDC is a progenitor population capable of differentiating along both luminal and myoepithelial lineages [10,11,14,15], or that tumor cells derived from a restricted progenitor population are capable of transdifferentiating, at least in terms of the expression of CK markers.

It has been suggested that progenitor cells capable of differentiating along both pathways exist that already express some of the genes characteristic of the basal, myoepithelial compartment [10,15,16], notably CK5/14. Indeed, several transgenic mouse models of mammary cancer have been developed that support this idea. Glukhova and colleagues used the CK5 promoter to overexpress a β-catenin variant, ΔN59Cat, that cannot be phosphorylated at a site necessary for normal turnover [17]. Hence, these mice accumulate transcriptionally active β-catenin in CK5-expressing cells, mimicking the effects of Wnt pathway stimulation. The nulliparous CK5. ΔN59Cat mice exhibit hyperplasia of the myoepithelial cell compartment of the mammary gland. However, multiparous transgenic animals exhibit a high incidence of mammary tumors, including both CK5^+^CK10^+ ^squamous cell carcinomas, and invasive carcinomas that express both CK5 and CK8. Another mouse model that supports this concept used the CK14 promoter to drive Cre-dependent recombination that resulted in the loss of two tumor suppressor genes, TP53 and BRCA2 [18]. Two-thirds of the double knockout females develop mammary tumors by one year of age that exhibit both luminal and myoepithelial differentiation. Thus, ample evidence exists that genetic alterations affecting CK5/14-expressing cells, presumably a progenitor population, can give rise to IDC that contain cells with characteristics of both the luminal and myoepithelial lineages.

We have recently described a transgenic mouse model in which the bovine CK5 promoter drives expression of the human ATF3 gene [19]. ATF3 is a transcription factor in the bZip family whose expression is induced during the response to DNA damage and other forms of cellular stress in a wide variety of tissues and cell types [20-22]. However, the physiological function of ATF3 is unknown, and its downstream transcription targets have not been extensively defined. These BK5.ATF3 mice exhibit several epidermal phenotypes, including a gross hyperplasia of the outer root sheath cells of the hair follicles, resulting in abnormal hair growth, and a milder hyperplasia of the interfollicular epidermis [19]. Additionally, aged transgenic mice (16 months) develop oral cavity neoplasias at high frequency including both squamous cell carcinomas, and a class of basal cell tumors with heterogeneous differentiation. We now describe several mammary phenotypes of heterozygous BK5.ATF3 mice, including the presence of cystic, squamous metaplasias in nulliparous female animals and a high incidence (67%) of mammary carcinomas with squamous features in parous females by one year of age.

## Methods

### BK5.ATF3 mice

As described previously [19], transgenic BK5.ATF3 mice were obtained by embryo injection in FVB/N mice. The construct for these experiments contained the human ATF3 cDNA sequence [23] in an expression vector containing the bovine keratin 5 promoter [24]. This promoter is active in basal epithelial cells in several tissues, including skin, thymus, and mammary gland (*vide infra*). Five independent lines that transmitted the transgene were established; four of these displayed an easily observable sparse hair phenotype. None of these lines showed significant neo-natal mortality, and only line 2 produced significantly fewer transgenic than non-transgenic progeny (4.1 ± 2.2 non-transgenic pups at weaning, 2.2 ± 2.0 transgenic pups at weaning, p = 0.00075). We have been unable to demonstrate transgene expression in the fifth line (line 6, no observable hair phenotype) in either skin or mammary gland, and this line was not studied further.

Mice were maintained in a light and temperature controlled room in an AAALAC-accredited facility, and given water and lab chow *ad libitum*. All experimental procedures were approved by the Institutional Animal Care and Use Committee.

Genotyping was performed using genomic DNA purified from tail snips as template. For most of these studies, a previously described PCR assay [19] was used. Alternatively, a real time PCR assay for the coding region of human ATF3 (Hs00231069_m1, Applied Biosystems, Foster City, CA) was utilized, with analysis on an Applied Biosystems 7900 HT. A positive control assay for an endogenous murine gene (Atf3, Gapdh or Rash) was also performed with each DNA sample.

### Histopathology and Immunohistochemistry

Tissues obtained at necropsy were fixed in buffered neutral formalin and paraffin embedded. Sections were prepared and stained (either with hematoxylin and eosin or for IHC) by the Tissue Processing Facility Core of the Center for Research on Environmental Disease. Tissue microarrays containing normal human mammary samples and breast cancer samples were the kind gift of Dr. A.J.P. Klein-Szanto (Fox Chase Cancer Center), or were purchased from Zymed (San Francisco, CA). For IHC, antigen retrieval was performed as described [19]. Binding of primary antibodies to tissue sections was visualized with a chromogenic substrate, using a secondary antibody coupled to horseradish peroxidase as described [19]. The sources of the primary antibodies were as follows: CK5, CK6, CK10, Covance (Berkeley, CA); CK8, Developmental Studies Hybridoma Bank (U. of Iowa, Ames, IA); Ki67, Dako (Carpinteria, CA); ATF3, ERα, ErbB2, Santa Cruz (Santa Cruz, CA). Polyclonal antibodies specific for inner root sheath keratins mIRSA2 and mIRSA3.1 [25] were generously provided by Dr. R.M. Porter (University of Cardiff). The blocking peptide for ATF3 IHC (sc-188p) was obtained from Santa Cruz (Santa Cruz, CA). IHCs for each marker were prepared from at least 10 mammary tumors obtained from 10 different animals. For quantitative analyses of cytoplasmic expression of CK10, over 1000 cells/tumor were evaluated in three micrographs prepared from each of the 10 tumors.

### Immunoblotting

The fourth and fifth mammary glands were obtained from virgin female mice, either non-transgenic FVB, or from BK5.ATF3, lines 1–4. Nuclear extracts were prepared using the NE-PER kit (Pierce). 60 μg of nuclear extract were electrophoresed on 4–20% SDS-polyacrylamide gels and transferred onto Immobilon-FL membranes (Millipore); RAW 264.7 whole cell lysate (Santa Cruz, sc-2211) was used as a positive control. Rabbit polyclonal anti-ATF3 or anti β-actin antibodies (Santa Cruz) and ECL Plus (Amersham) were used to detect the respective proteins.

### Statistics

The incidence of squamous metaplastic lesions in nulliparous females was compared between transgenic and non-transgenic animals by Fisher's exact test. Differences in survival between genotypes and groups were analyzed by logrank test. Quantitative differences in litter size and transmission of the transgene were analyzed by t-test.

## Results

### Mammary glands show aberrant growth in virgin BK5.ATF3 animals

As described elsewhere [19], transgenic BK5.ATF3 animals exhibit several epidermal phenotypes, including sparse hair, marked hyperplasia of the outer root sheath cells of the hair follicle, inability to complete the hair cycle, and mild chronic hyperplasia of the interfollicular epidermis. Late in life, hyperplasia, dysplasia and frank neoplasia is seen in the oral cavity, but no epidermal tumors have been observed.

**Figure 1:**
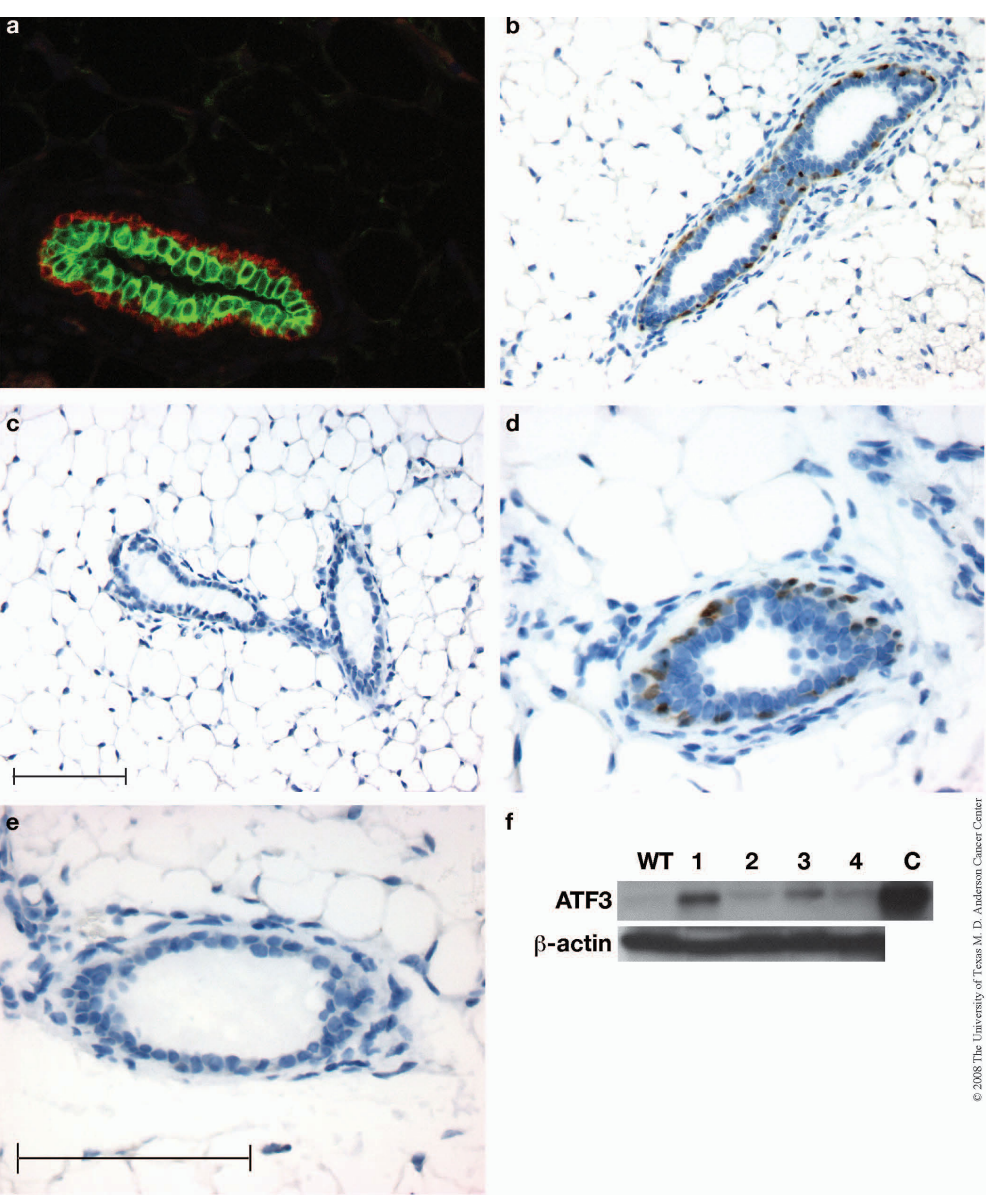
**ATF3 expression in myoepithelial cells of BK5.ATF3 mice**. (a). A paraffin section (4 μm) of a mammary gland from an 8 week old BK5.ATF3 virgin female was stained with fluorescently labeled antibodies to CK5 (Texas Red, red) and CK8 (FITC, green) and examined by fluorescence microscopy. (b-e). Sections were also treated with a primary antibody to ATF3, then stained using horseradish peroxidase-coupled secondary antibody and a chromogenic substrate that produces a brown stain, and counterstained with hematoxylin. Mammary gland from a BK5.ATF3 transgenic female (b,d) was compared with a non-transgenic littermate (c,e). Scale bars = 100 μm; scale bar in panel c applies to panels b and c, scale bar in panel e applies to panels d and e. (f). Nuclear extracts were analyzed for ATF3 protein expression by immunoblotting as described in Materials and Methods. Equal amounts of protein from mammary glands of non-transgenic mice (lane WT) or BK5. ATF3 mice of line 1 (lane 1), line 2 (lane 2), line 3 (lane 3), or line 4 (lane 4) were analyzed. As a positive control, an extract from RAW 264.7 cells was analyzed (lane C). β-actin was used as loading control. An ATF3-specific band was detected in the 20–25 KD size range in the BK5.ATF3 mammary gland extracts and in the positive control extract; this band was barely detectable in extracts from non-transgenic mice (lane 1). The ATF3 band was abolished by preincubation with the ATF3 blocking peptide (not shown).

The ducts of the mammary gland are composed of two layers of epithelial cells: an outer layer of myoepithelial cells that express CK5 (Figure 1a, red) and an inner layer of luminal cells that express CK8 (Figure 1a, green). Thus, the myoepithelial cells of BK5.ATF3 mice might be expected to express ATF3, and this was confirmed by IHC in mammary gland sections of 8 week old BK5.ATF3 mice (Figure 1b,d). The immunohistochemically-detected ATF3 protein appeared to be concentrated in the nuclei of the myoepithelial cells, as expected for a transcription factor. No ATF3 expression was detectable by IHC in the mammary glands of wild type littermates (Figure 1c,e), although the antibody used cross-reacts with ATF3 of both human and murine origin [26]. This is not unexpected, since ATF3 expression is typically only detected after treatments that induce DNA damage or other conditions of cellular stress [19-22]. Analysis of nuclear extracts by immunoblotting revealed strong expression of the protein in BK5.ATF3 line 1 (Figure 1f, lane 1), weaker expression in lines 2–4 (lanes 2–4) and barely detectable expression in non-transgenic animals (lane WT), presumably due to endogenous ATF3.

**Figure 2:**
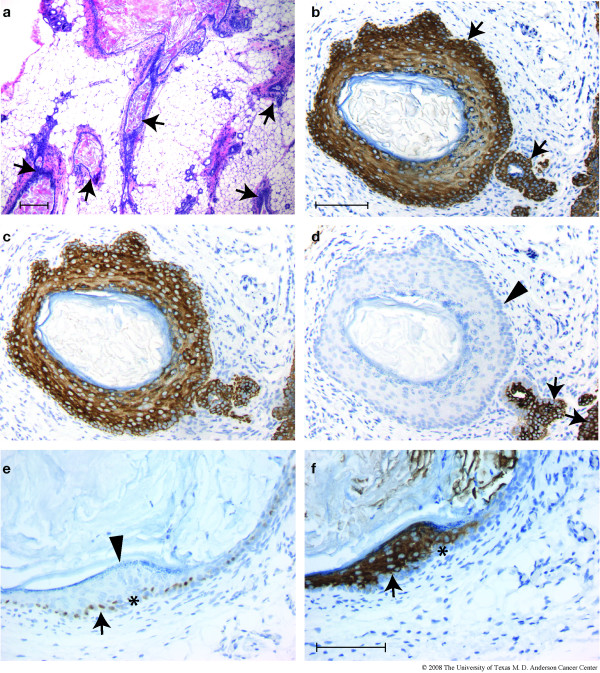
**Squamous metaplasia in virgin transgenic animals**. Mammary glands from 21 nulliparous, BK5.ATF3 line 1 mice between 14 and 32 weeks of age and 18 age-matched, nulliparous non-transgenic littermates were examined histologically and by IHC. Transgenic mice from three other BK5.ATF3 lines that express the transgene were also examined (Table 1). (a) A low power view of multiple squamous metaplastic lesions in a single gland of a BK5.ATF3 line 4 female. (b-f) Two typical, cystic lesions from BK5.ATF3 line 1 mammary glands are shown, stained for (b) CK5; (c) CK6; (d) CK8; (e) ATF3; (f) CK10. Scale bar in a = 200 μm; scale bar in b = 100 μm, applies to panels b, c, and d; scale bar in f = 100 μm, applies to panels e and f.

When mammary glands were examined in somewhat older virgin females (14–32 weeks), numerous dysplastic lesions were found in all four independently derived BK5.ATF3 transgenic lines. For many of the affected glands, multiple ducts exhibited squamous metaplasia (Figure 2a, arrows), and a sizeable fraction of the gland was abnormal. The lumens of the affected ducts were filled with keratin, and, depending on the plane of the section, often appeared as cysts. In BK5.ATF3 line 1, squamous metaplastic lesions were seen in 15 of 21 transgenic females examined; no lesions were seen in 18 non-transgenic animals examined (Table 1). This difference in incidence is statistically significant (Fisher's exact test, p = 2.84 × 10^-6^). The other three transgenic lines examined exhibited squamous metaplastic mammary lesions in from 46.2 to 66.7% of the animals examined (Table 1); in all cases the difference between transgenic and non-transgenic animals was statistically significant (Fisher's exact test, p < 0.001). In transgenic lines 1 and 2, over 20% of the glands contained one or more lesions; this fraction was somewhat lower in the other two lines (Table 1). Expression of CK5 in these lesions was most intense in the outermost, basal-like layer of cells, and in all lesions multiple layers of CK5-immunoreactive cells were seen (arrow, Figure 2b). Cytokeratin CK6, not normally observable in mammary ducts of post-pubescent animals, was strongly expressed throughout the lesions (Figure 2c). Expression of CK8 within the lesions was variable; when present, the cells stained intensely (Figure 2d, arrows), but other areas appeared to completely lack CK8 expression (Figure 2d, arrowhead). The basal-like outer layer of cells (denoted by an asterisk in Figure 2e &2f) routinely expressed nuclear ATF3 (Figure 2e, arrow) and the Ki67 proliferation marker (data not shown). Strong staining for the interfollicular epidermal keratin CK10 [27] was also seen in some lesions (Figure 2f, arrow), and when present was noted to be expressed supra-basally. An additional marker of squamous differentiation, keratohyaline granules, could be seen in most lesions (Figure 2e, arrowhead).

**Table 1:** Squamous metaplasia in nulliparous females

Line	Non-transgenic	Line 1 TG	Line 2 TG	Line 3 TG	Line 4 TG
No. of Animals Examined	18	21	9	13	13
No. of Animals with metaplasia	0	15	9	6	6
Incidence of Metaplasia	-	0.714	0.667	0.462	0.462
No. of Glands Examined	108	151	84	116	107
No. of Squamous Metaplasias	0	32	23	6	11
Fraction of Glands with					
Metaplasia	-	0.21	0.27	0.05	0.1

### Mammary tumors develop in parous BK5.ATF3 mice

Initially, BK5.ATF3 females were used as breeders to maintain the lines by mating with non-transgenic FVB/N males. However, litter sizes at weaning tended to be smaller for transgenic dams (6.4 ± 2.2 pups) compared to non-transgenic dams (8.5 ± 2.9 pups), the pups had lower weights, and the pups (both transgenic and non-transgenic) were apparently unable to suckle from several teats (data not shown). Because of these problems, it was decided to retire the transgenic dams, and maintain the line by mating BK5.ATF3 males to FVB/N dams. Unexpectedly, three of three retired BK5.ATF3 line 1 female breeders developed large mammary tumors between 6 months and 1 year of age.

**Figure 3:**
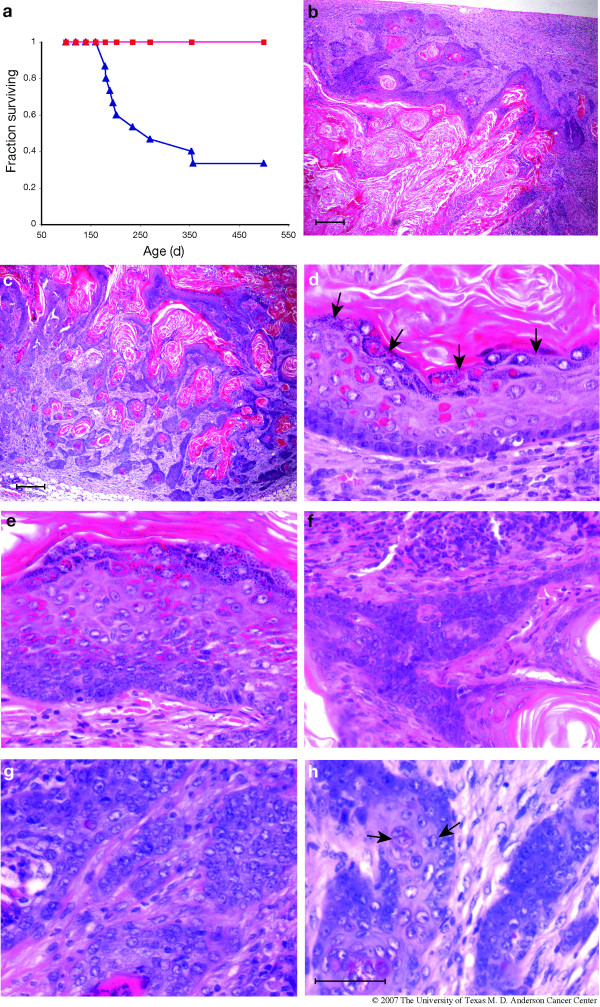
**Mammary tumors in parous BK5.ATF3 mice**. (a). Female wild-type mice (n = 12, closed squares) or BK5.ATF3 mice (n = 15, closed triangles) were allowed to mate and raise pups twice between 6 and 24 weeks of age, then observed until 16 months of age. Mice were euthanized when tumors reached 1.5 cm. Groups of nulliparous wild type (n = 20) and BK5.ATF3 (n = 13) females were also maintained for 16 months (data not shown); survival of both of these groups was 100% at the conclusion of the experiment. Sections of two representative tumors arising in BK5.ATF3 mice are shown at low power (b,c; scale bar = 200 μm); sections from five different tumors are shown at higher power (d-h; scale bar = 50 μm). KC, keratinaceous core.

To better understand this finding, cohorts of non-transgenic FVB/N and BK5.ATF3 line 1 females were either maintained as virgins for 16 months, or mated and allowed to raise pups to weaning twice between the ages of 6 and 13 weeks. All animals were monitored weekly for palpable mammary masses and sacrificed when such masses reached 1.5 cm in diameter or when the animals became moribund. Surviving animals were sacrificed at 16 months. The survival of BK5.ATF3 and non-transgenic, biparous female mice is shown in Figure 3a. No suspicious masses were observed in the biparous wild-type mice (red squares), and none were seen in virgin non-transgenic or BK5.ATF3 mice during the 16 month observation period (data not shown). Mammary tumor incidence reached 67% within the biparous BK5.ATF3 group (blue triangles) by 12 months of age. Logrank analysis of the survival curves indicated a significant difference between the biparous BK5.ATF3 group and the biparous non-transgenic group (p = 0.0005).

At necropsy, the masses were found to be confined to the mammary glands. About 80% of the tumor-bearing animals (12 out of 15; this includes 3 transgenic tumor-bearing mice examined after the original survival study was completed) had masses in more than one gland. All mammary masses occurring in the biparous BK5.ATF3 mice were examined by hematoxylin and eosin staining of paraffin sections and found to be carcinomas with squamous differentiation; examples of the histopathology are shown in Figure 3b–h. The bulk of the tumors consisted of large, cystic lesions with a well-defined epithelium surrounding keratinaceous debris, dead cells and infiltrating inflammatory cells (Figure 3b &3c). Significant atypia and dysplasia could be observed in all tumors. In some areas, the epithelial component was relatively well organized (Figure 3d), with a basal layer one-two cells in thickness, and suprabasal components that mimicked squamous differentiation, including the appearance of keratohyaline granules adjacent to the keratinaceous core (arrows). In other areas, the epithelial component became more hyperplastic (Figure 3e), more anaplastic with increasing signs of atypia (Figure 3f), and finally transitioned into frank neoplasia, exhibiting features of squamous cell carcinoma (Figure 3g &3h). Atypia was noted in all layers (Figure 3h), and atypical, enlarged nuclei were evident (arrow). The stroma of the tumors was extremely cellular, with abundant inflammatory infiltrates. Signs of stromal invasion were seen in most lesions (for example, Figure 3g).

**Figure 4:**
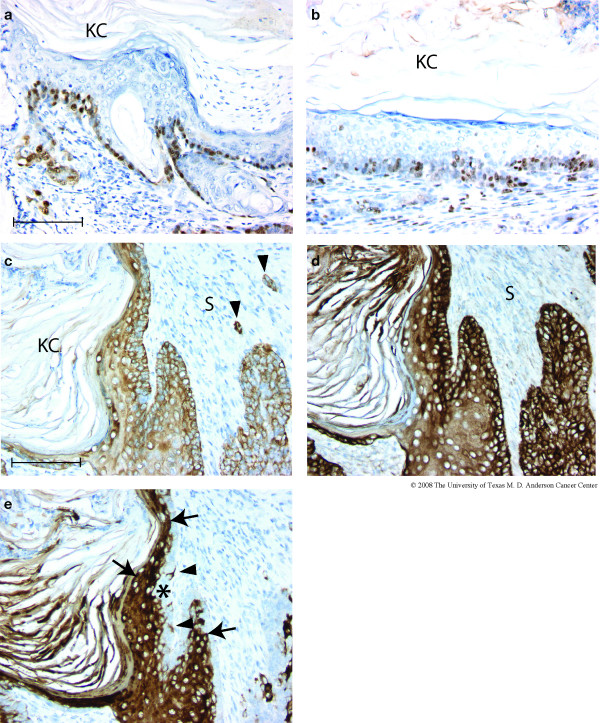
**ATF3, Ki67 and cytokeratin expression in BK5.ATF3 mammary tumors. **Immunohistochemistry of representative tumors arising in BK5.ATF3 mice utilizing primary antibodies directed against ATF3 (a), Ki67 (b), CK5 (c), CK8 (d), and CK6 (e). Scale bars = 100 μm. KC, keratinaceous core; S, stroma.

Immunohistochemistry indicated that the outermost, basal layers of tumor cells (that is, furthest removed from the keratinaceous core) typically exhibited nuclear expression of the ATF3 transgene (Figure 4a), and were enriched for dividing cells as shown by expression of the Ki67 proliferation marker (Figure 4b). CK5 immunoreactivity, characteristic of basal epithelial cells, was high in both basal and supra-basal layers of the metaplastic lesions virtually throughout all tumors examined (Figure 4c). In rare areas, CK5 expression was confined to the most basal cell layer (not shown). Stromal components (S) were not immunoreactive for CK5 except for scattered "nests" of tumor cells (Figure 4c, arrowheads). Interestingly, CK8, characteristic of luminal mammary epithelial cells, was also expressed in both basal and supra-basal cells in 14 of 14 tumors examined (Figure 4d). Co-expression of CK5 and CK8 throughout all layers of the epithelial component was the predominant expression pattern in 9 of these tumors. In the remaining 5 tumors, approximately half of the epithelial component of the tumors showed this expression pattern. The remaining areas either exhibited no expression of CK8, or expression was much stronger in basal cells than in supra-basal cells. The widespread, intense expression of CK8 confirms that the tumors arise from mammary epithelium.

**Figure 5:**
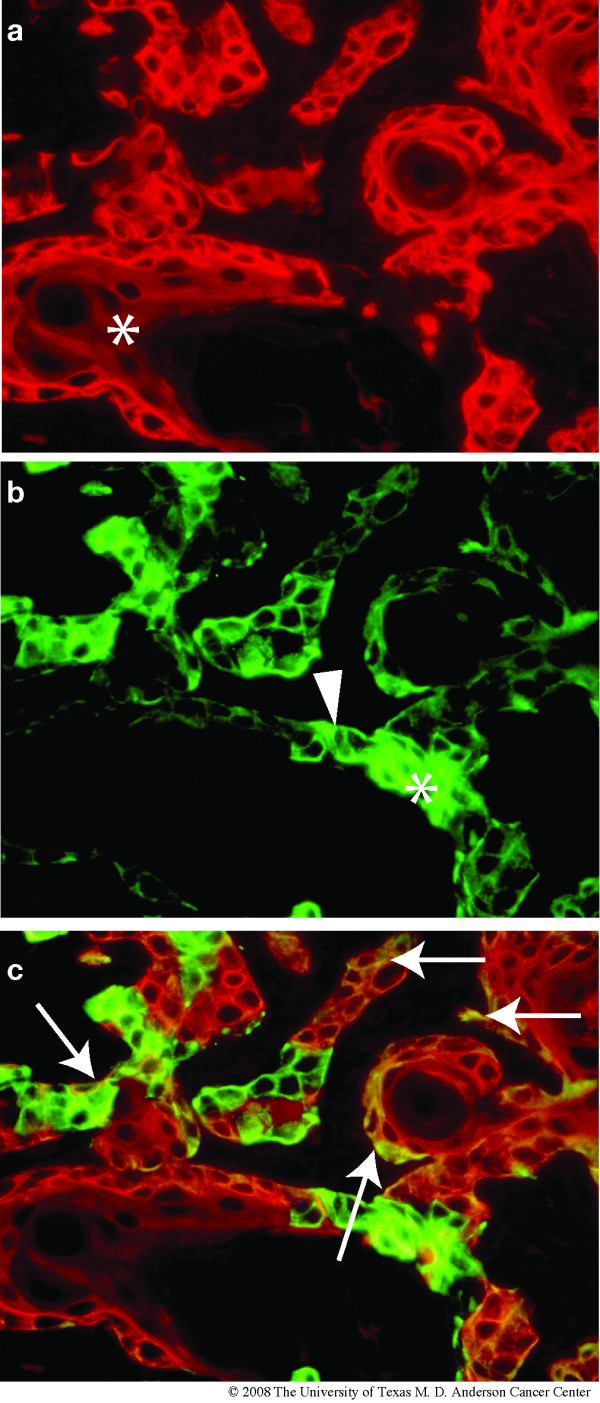
**Co-expression of CK5 and CK8 in tumor cells**. A paraffin section of a mammary tumor arising in a BK5.ATF3 parous female was stained with fluorescently labeled antibodies to CK5 (Texas Red, red) and CK8 (FITC, green) and examined by fluorescence microscopy. (a), CK5 (b), CK8 (c), merged image.

In most human breast tumors that express both CK5 and CK8, these two cytokeratins are generally confined to distinct sub-populations of tumor cells. Thus, our finding from the standard IHC studies that the majority of tumor cells in the ATF3-induced murine tumors stained for both CK5 and CK8 was somewhat unusual. To confirm this finding, we repeated the IHC analysis using anti-CK5 antibody tagged with a red fluorescent dye, and anti-CK8 antibody tagged with a green fluorescent dye, and analyzed the results by fluorescence microscopy. Figure 5, panels a and b shows a representative portion of a BK5.ATF3 mammary tumor photographed with filters for the individual dye-tagged antibodies, with the two signals merged in panel c. Groups of fluorescently-labeled tumor cells in a background of unlabeled stroma were seen with both antibodies, and the merged image indicates that these groups are in many cases approximately co-extensive. Within a group, the fluorescent intensities in individual cells varied over a broad range for both antibodies. For example, in the tumor cell island marked with a white asterisk in panel b, highly fluorescent and barely fluorescent CK8-labeled cells were seen, and the physical extent of the CK5 labeling in this island suggests the presence of CK5-stained cells that do not stain for CK8 (asterisk in panel a). Conversely, the white arrowhead in panel b marks a cell that appeared to be CK8-positive and CK5-negative. However, the most common pattern seen was expression of both markers in individual cells, albeit at different levels of intensity. This was particularly evident in the appearance of yellow cells (white arrows) in the merged image of panel c. The predominant pattern of co-expression of cytokeratins characteristic of both the myoepithelial lineage (CK5) and the luminal lineage (CK8) suggests that the tumors may arise from progenitor cells capable of contributing to both major lineages.

Cytokeratin CK6 is generally not found in adult ductal mammary gland epithelium, but has been suggested to be a marker of mammary progenitor cells [14]. Indeed mammary tumors with phenotypic characteristics similar to those described here have been suggested to arise from mammary progenitor or stem cells [14]. Essentially all cells in the suprabasal layers of the BK5.ATF3 mammary tumors clearly expressed CK6 (Figure 4e, arrows). However, CK6 expression was generally absent in the basal, rapidly dividing cell layer (Figure 4e, asterisk), which is expected to contain the least differentiated, most "primitive" cells. Scattered cells within the basal layers did exhibit CK6 expression (Figure 4e, arrowheads). It is worth noting that supra-basal expression of CK6 is characteristically found in hair follicles [28].

**Figure 6:**
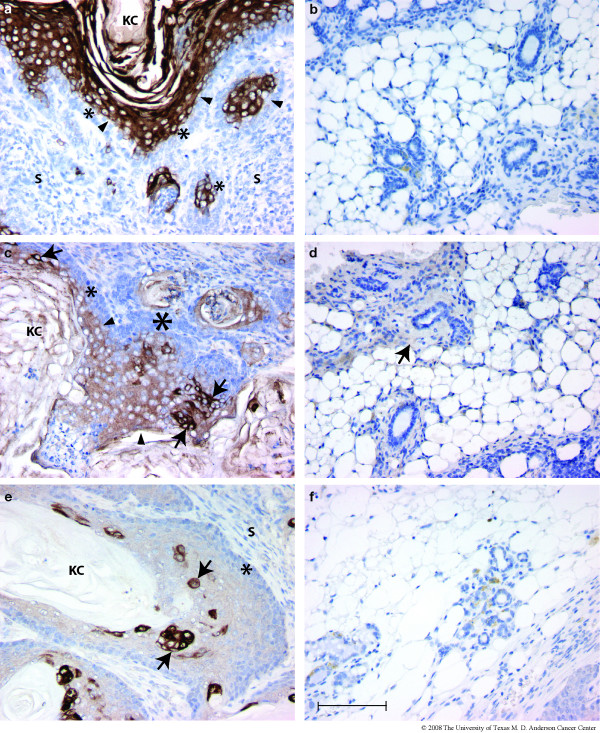
**Deregulation of cytokeratin expression in BK5.ATF3 mammary tumors. **Immunohistochemistry of representative mammary tumors arising in BK5.ATF3 mice (a,c,e) or uninvolved mammary ducts of tumor-bearing animals (b,d,f) was performed with primary antibodies to CK10 (a,b), mIRS.a2 (c,d) and mIRS.a3.1 (e,f), and stained with the chromogenic substrate. Scale bar = 100 μm. KC, keratinaceous core; S, stroma.

Further analysis of the BK5.ATF3 mammary tumors revealed deeper anomalies in cytokeratin expression that are not easily explained solely by mammary stem cell origin. Because the tumors exhibit squamous differentiation, the possibility existed that epidermal differentiation markers would be expressed in the tumors. As basal keratinocytes differentiate and move into the suprabasal layers of the epidermis, cytokeratin expression shifts from CK5 and CK14 to CK1 and CK10, two epidermal-specific keratins [27]. Analysis of ATF3-induced mammary tumors revealed widespread expression of CK10 in 10 of 10 tumors examined (Figure 6a). On average, about 46.6% (range 38–61%, standard deviation 6.3%) of the tumor cells expressed CK10. Generally, CK10 expression was not seen in basal cells (indicated in Figure 6a by asterisks), but was confined to the suprabasal layers (Figure 6a, arrowheads). Uninvolved mammary ducts of tumor-bearing animals were generally negative for CK10 expression, although scattered light background staining of stromal elements was seen (Figure 6b). We also looked for markers of hair follicle differentiation. Type I and type II keratins that are apparently only expressed in the supra-basal inner root sheath cells of hair follicles have been described [25]. IHC analysis with antibodies specific for the inner root sheath-specific type I keratin mIRSa.2 showed widespread, medium intensity staining of supra-basal tumor cells in 10 of 10 tumors examined (Figure 6c, arrowheads). Generally, the basal-most 2–3 cell layers (Figure 6c, asterisks) were unstained. The generalized, supra-basal staining was somewhat more intense than background staining seen in stromal elements of uninvolved, adjacent mammary gland tissue with this antibody (Figure 6d, arrow); no unequivocal staining of ductal epithelial cells was seen in uninvolved ducts. In addition to this generalized, medium intensity staining, scattered single cells and groups of cells stained intensely for mIRS.a2 in all tumors examined (Figure 6c, arrows). A second inner root sheath-specific type I cytokeratin, mIRS.a3.1, gave essentially the same results in 10 of 10 tumors examined (Figure 6e). The generalized, supra-basal staining pattern was weaker, but strong expression in scattered groups of tumor cells was seen in all tumors examined, comparable to the mIRS.a2 staining intensity (Figure 6e, arrows). Again, apparent background staining in uninvolved mammary ducts was seen in scattered stromal elements, and no unequivocal staining of ductal epithelium was seen (Fig. 6f). Thus, not only differentiation markers of two mammary cell lineages, but also differentiation markers of several epithelial lineages of the epidermis are expressed in these tumors. This suggests that over-expression of ATF3 results in fundamental alterations in the differentiation status of the affected tumor cells, including expression of markers characteristic of multiple tissues and epithelial lineages.

**Figure 7:**
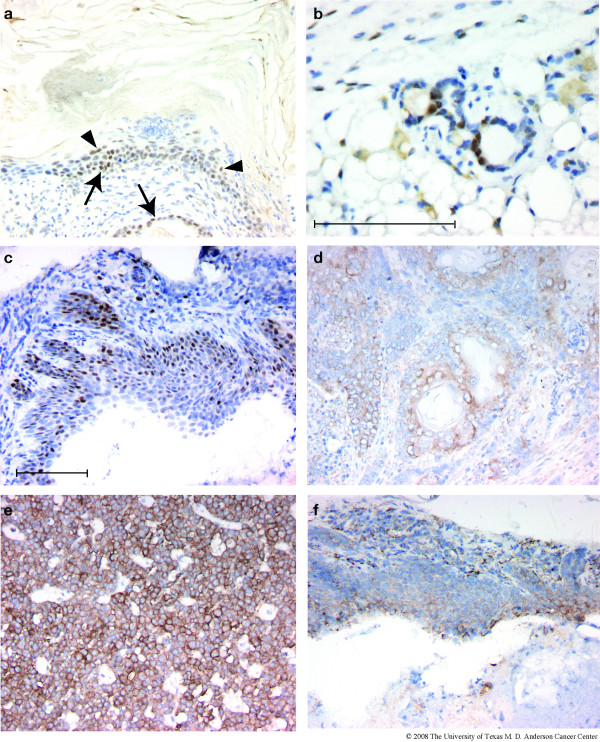
**Expression of ERα and ErbB2 in BK5.ATF3 mice**. Immunohistochemistry for ERα (a,b,c) or for ErbB2 (d,e,f) was performed with representative sections from mammary tumors arising in BK5.ATF3 mice (a,d), from a normal appearing mature mammary gland (b), from a mammary tumor that developed in an MMTV.neu transgenic animal (e), or from squamous metaplastic lesions (c, f) that occurred in the mammary glands of nulliparous BK5.ATF3 females. Scale bar = 100 μm.

Several markers commonly used to classify human breast tumors were analyzed in these tumor specimens to begin to understand the genesis of the ATF3-induced tumors. Expression of ERα is an important prognostic factor in human breast cancer: tumors that are ERα^+ ^have a more favorable prognosis. Over 90% (13 of 14 tumors examined) of the mammary tumors that developed in parous BK5.ATF3 female animals showed significant nuclear expression of ERα (Figure 7a). In about 40% of the tumors, expression was widespread, with apparent expression in both basal (arrows) and supra-basal (arrowheads) tumor cell nuclei. In the remaining tumors, expression was high in most regions of the tumor, but other regions were negative for ERα. Within ERα^+ ^regions of ten tumors examined in detail, an average of 74% of the cells expressed ERα (range 54–88%). In uninvolved regions of the mammary gland adjacent to the tumors, 50–60% of the ductal cells were ERα^+ ^(Figure 7b). In contrast, no tumors examined were markedly positive for ErbB2, a well-known mammary oncogene that is often amplified and overexpressed in human breast tumors. No expression was seen in 4 of 10 tumors examined, and the remaining tumors were marginally positive, with less than 10% of the tumor cells stained. As seen in Figure 7d, even in marginally positive tumors ErbB2 staining was relatively weak (compare with staining in a tumor derived from a transgenic MMTV.neu mouse, Figure 7e). Interestingly, both of these markers were expressed at moderate to high levels in most of the metaplastic lesions seen in mammary glands of virgin BK5.ATF3 females (Figure 7c,f).

### Expression of ATF3 in human breast tumors

Almost 70% of human breast tumors exhibit amplification of parts of the q arm of chromosome 1 [29-32]. The ATF3 gene is located at 1q32.1, in a region often overrepresented as detected by comparative genomic hybridization studies. Direct measurements of ATF3 copy number by quantitative PCR indicated some amplification in ~80% of human breast tumors [33]. As part of a more extensive study of gene expression signatures in human breast tumors, expression of ATF3 was measured in a series of 28 early (stage 1–2) breast tumor samples. Although ATF3 expression is barely detectable in normal mammary cells, expression at high levels (at least 4-fold higher than the mean of all genes assayed) was detected in many of the samples (manuscript in preparation). Furthermore, overexpression of ATF3 protein in 50% of human breast tumors has recently been reported by Hai and co-workers [33].

**Figure 8:**
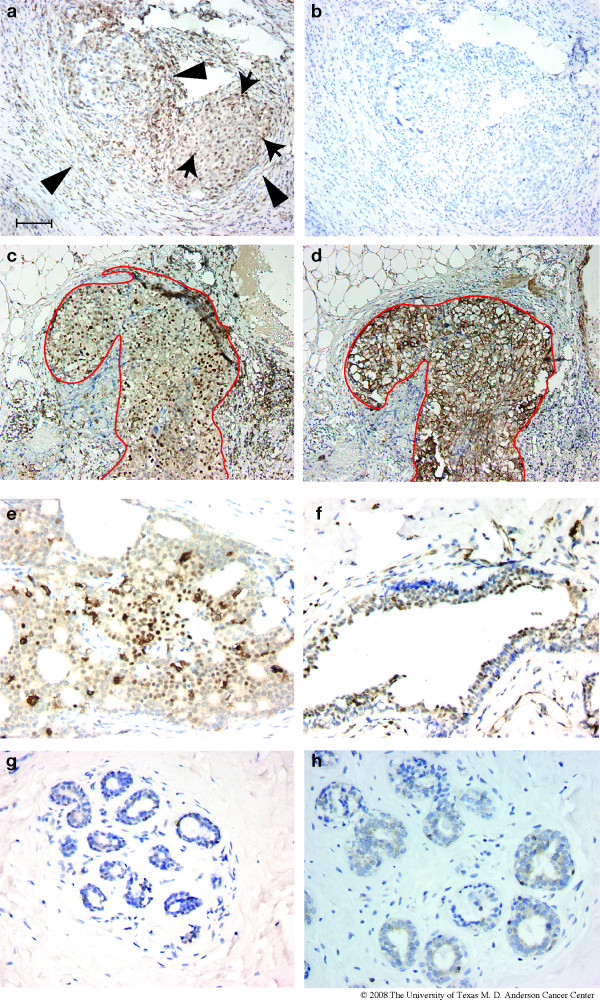
**Expression of ATF3 in human mammary tumors**. Paraffin sections from human invasive ductal carcinomas that were less than 2 cm at presentation were examined by immunohistochemistry with an antibody directed against ATF3 (a-c,e,f). In (b), a blocking peptide for ATF3 (sc-188p, Santa Cruz) was added prior to the ATF3 antibody; the same region of the tumor is shown in (a) and (b). Panels a, c, and e represent tumors from three different patients. An adjacent section to that shown in (c) was analyzed for CK5 expression, and the corresponding region of the tumor is shown in (d). (f) shows a hyperplastic duct adjacent to an ATF3-positive tumor. (g-h), paraffin sections from normal mammoplasty tissues were examined by IHC with the same ATF3-specific antibody. Scale bar = 100 μm.

To determine whether the overexpression detected at the RNA level and by immunoblotting was reflected in protein expression levels in the epithelial component of human tumors, paraffin-embedded sections were obtained from 18 early breast tumors, similar to those studied at the RNA level, and analyzed for ATF3 protein by IHC. Sections derived from non-tumorous mammary tissue (mammoplasty specimens) were also analyzed. Adjacent sections were analyzed in parallel by IHC for CK5 or after staining with hematoxylin and eosin to better define the cellular elements of the tumors. Many of the tumors exhibited low levels of cytoplasmic expression of ATF3, as well as scattered positive nuclei (Figure 8a, arrows). In addition, almost all of the tumors (17 of 18) exhibited diffuse staining of stromal elements, including adipocytes, fibroblasts and infiltrating inflammatory cells; a representative view is shown in Figure 8a (arrowheads). When a blocking peptide was added prior to addition of the ATF3 primary antibody, staining of both stromal and epithelial components was abolished, confirming the specificity of IHC staining (Figure 8b). About 40% of these tumors also contained significant, well-defined regions (i.e., foci of malignant epithelial cells surrounded by stromal elements and containing at least 200 tumor cell nuclei in the section examined) that exhibited strong nuclear staining in at least 30% of the nuclei. An example is shown in Figure 8c. To identify the epithelial portion of the tumor, an adjacent section was stained for CK5 (Figure 8d), and the observed boundaries of the epithelial components are indicated by a red line in both panels. In many cases, the regions with high levels of staining appeared to be ductal carcinoma *in situ *(DCIS) (e.g., Figure 8c and 8e), located within the tumor. In all cases examined, the tumor regions that exhibited ATF3 nuclear staining were also positive for cytoplasmic CK5 (e.g. Figure 8d) and CK8 (data not shown). None of the tumors examined appeared to have strong nuclear ATF3 expression in all tumor cells. In addition, clear nuclear ATF3 expression was seen in hyperplastic ducts located around the periphery of several tumors (Figure 8f); both basal and supra-basal cells in these abnormal ducts were immunoreactive for ATF3.

None of the normal mammary tissues examined exhibited staining for ATF3 in the stromal elements, and the staining of epithelial nuclei was generally much lower than in the ATF3-positive tumors (Figure 8g–h). In some cases, diffuse cytoplasmic staining was seen in scattered acini. A small fraction of large, apparently normal, ducts in both normal mammoplasty tissues and in tumor-adjacent normal tissue exhibited strong nuclear ATF3 expression in the basal layers (not shown).

To confirm the overexpression of ATF3 protein in human mammary tumors, tissue microarrays were obtained and analyzed by the same method. Overall, a total of 127 cores representing mammary tumors and 13 normal mammary glands were analyzed. Strong nuclear expression of ATF3 in the mammary tumor cells was detected in 18% of the tumors overall; none of the normal samples in these arrays showed nuclear ATF3 expression.

## Discussion

The finding that overexpression of ATF3 in basal epithelial cells of the mammary gland induces mammary tumorigenesis was unexpected. At the outset of this project, there were no reports clearly identifying ATF3 as an oncogene, although several publications suggested a role in tumorigenesis [34-36]. Our initial description of the BK5.ATF3 mouse model [19] reported a high incidence (~70%) of oral carcinomas in older transgenic mice of both genders, and was the first evidence clearly suggesting that ATF3 may act as an oncogene. In addition, the hair follicles in these mice exhibited extreme hyperplasia in the outer root sheath, and mild hyperplasia in the interfollicular epidermis, including overexpression of CK6. Recently, Hai and co-workers [33] also suggested that ATF3 is an oncogene, based primarily on widespread overexpression of ATF3 protein in human breast tumors, detected by immunoblotting. We have confirmed this finding at the mRNA level (unpublished data), but our IHC analysis (Figure 8) indicates that some of this overexpression is confined to stromal cells rather than carcinoma cells.

In the tumor experiment reported in Figure 3, all of the tumors arose by one year of age, and no tumors were seen in a total of 32 non-transgenic and 13 nulliparous BK5.ATF3 females observed for 16 months. This makes it extremely unlikely that the observed tumors are related to the background pituitary adenoma-induced mammary lesions that arise late in life in some colonies of FVB/N mice [37,38]. In a previous study ([19] and unpublished results) in which sagittal sections of the head were examined (2 in each animal), we identified a single pituitary adenoma in 43 female FVB/N examined at 16 months. Furthermore, the results of an independent tumor study with BK5.ATF3 mice carrying a β-galactosidase reporter gene were very similar to the results shown in Fig. 3 (ms. in preparation); no background mammary tumors were seen in 10 non-transgenic animals at 16 months. Since the transgene expression is not organ-specific, it is possible that the effects on the mammary gland are indirect, due to overexpression in another tissue. We have not seen transgene expression in several other organs that express CK5, notably forestomach, thymus, and pancreas. Overexpression is seen in epidermis and oral epithelia [19]. Hyperplasia is the major phenotype seen in skin, with no development of neoplasia. Carcinomas do develop in the oral cavity, but the onset of tumorigenesis is delayed relative to the appearance of mammary tumors. This delay, along with the absolute dependence of mammary tumorigenesis on parity, make it unlikely that an indirect effect due to skin or oral expression of ATF3 plays a major role in mammary tumorigenesis. We therefore conclude that overexpression of ATF3 in the mammary gland is a contributing factor for mammary tumorigenesis in this transgenic model.

Indeed, the biological roles played by ATF3 remain poorly understood. ATF3 expression is a ubiquitous feature of the response to DNA damage and other cellular stressors [21,22,39,40], and several downstream targets have been identified [33,41-48] without, however, providing much insight into the physiological function of the gene. The ATF3 response to DNA damage appears to be regulated by p53 [39,49] and by stimulation of the MAPK pathway [39,50], but it is a self-limiting response in that the ATF3 protein acts as an inhibitor of its own promoter [47]. There is also evidence for down-regulation of p53 by ATF3, blocking apoptosis of cardiac myocytes treated with doxorubicin [51] and human umbilical cord endothelial cells treated with tumor necrosis factor-alpha [52]. Additionally, ATF3 appears to be growth stimulatory in hepatocytes, dorsal root ganglion neurons, and classical Hodgkin lymphoma cells [53-55]. In contrast to these pro-growth/survival effects, ATF3 has also been positively associated with growth arrest or apoptosis in human umbilical cord endothelial cells, pancreatic beta-cells, chondrocytes and mouse embryonic fibroblasts [35,56-59]. Involvement of ATF3 in the normal function of the unstressed mammary gland has not been reported.

Some insight into possible effects of ATF3 overexpression has been provided by studies of its interactions with other transcription factors. As a member of the bZip family of transcription factors, ATF3 can form heterodimers with other family members through the leucine zipper domain, as well as forming homodimers. Known binding partners include cJun, JunB, CEBP-γ and Ddit3 [60]. It has been reported that ATF3 homodimers act as repressors of transcription [23,46], while heterodimers with cJun and JunD can activate transcription [42,61,62]. In addition, recent studies have identified physical and functional interactions of ATF3 with two other transcription factors, p53 [48,63] and Smad3 [43], that are leucine zipper-domain independent. However, physiological roles for these heterodimers or for ATF3 homodimers in mammary gland are unknown.

Our working hypothesis is that overexpression of ATF3 in a CK5-expressing stem or progenitor cell (postulated to be capable of differentiating along either myoepithelial or luminal pathways [10,15,16]) interferes with important cellular signaling pathways, either by a direct transcriptional effect on a normal ATF3-target gene or by sequestering other transcription factors. As noted above, these may include p53, Smad3, cJun, Ddit3 and other known binding partners of ATF3. The transcriptional repression ability of ATF3 may then effectively abrogate the function of one or more of these pathways, and thereby initiate the process of tumorigenesis. The absolute dependence of ATF3-induced tumorigenesis on parity (see Figure 3) suggests that the growth-promoting stimuli that the mammary gland responds to during pregnancy and lactation may promote the growth of initiated stem or progenitor cells in the transgenic glands, eventually leading to malignant carcinoma.

The squamous differentiation characteristic of ATF3-induced tumors is phenotypically exceedingly similar to tumors produced in several transgenic models in which the Wnt/β-catenin pathway is stimulated. These models include transgenes expressed in the basal cell compartment [17] and transgenes expressed primarily in the luminal cell compartment due to the use of the MMTV promoter [14,64-68]. In particular, the characteristics of the BK5.ATF3 model, including the squamous differentiation phenotype of the tumors, the expression of CK10, and the dependence of tumorigenesis on parity, are very similar to the CK5.ΔN59cat model described by Glukhova et al. [17]. Two models explaining the squamous metaplastic phenotypes of the Wnt/β-catenin tumors have been proposed. Based on expression of markers of more "primitive" stem or progenitor cells (CK6 and Sca-1), Varmus and colleagues [14] suggested that the target cell population must be a very primitive stem cell. Alternatively, it has been suggested [65,66] that the phenotype was due to "transdifferentiation" to the epidermal lineage. The carcinomas that develop in multiparous BK5.ATF3 mammary glands express a wide range of marker proteins, characteristic not only of both basal and suprabasal compartments of the mammary gland (CK5 and CK8, respectively), but also the suprabasal compartments of the interfollicular epidermis (K10) and the hair follicles (mIRSa.2, mIRSa.3.1). Expression of both basal and suprabasal markers suggests strongly that the target population for tumorigenesis contains CK5-expressing, multi-potential progenitor cells capable of giving rise to both basal and suprabasal components. A model that combines components of both the Varmus and Hennigshausen models would be that one of the effects of ATF3 overexpression in these progenitor cells would be to "erase" the developmental specifications that mark this population as mammary, and allow them to differentiate as either mammary or epidermal, the decision presumably being stochastic. The observed pattern of expression of the hair follicle cytokeratins (Figure 6) is consistent with such a model, in that the aberrant markers (mIRSa.2, mIRSa.3.1) are generally expressed in localized foci of cells, as would be expected for a clone of tumor cells descended from a single progenitor cell that became specified for a particular epithelial compartment stochastically.

The finding of high levels of nuclear expression of ATF3 in a subset of human breast tumors (Figure 8) raises the possibility that this transgenic model may provide clues as to the genesis of at least some human cancers. Interpretation of these results is complicated by the simultaneous expression of ATF3 at high levels in stromal components of these tumors (Figure 8), and by the fact that expression within the epithelial cell compartment is by no means universal within a given tumor. Thus, it is also possible that in both stromal and epithelial tumor components, ATF3 expression is part of an ongoing response to stressful conditions existing within the tumor microenvironment. However, ATF3 expression is seen more frequently in DCIS components of the tumors, and in hyperplastic ducts adjacent to ATF3-positive tumors. Thus, it is conceivable that aberrant nuclear ATF3 expression is involved in the early, rather than the late, stages of malignant transformation.

## Conclusion

Overexpression of ATF3 in CK5-expressing murine mammary epithelial cells results in the development of squamous metaplastic mammary lesions in nulliparous mice, and in mammary tumorigenesis in biparous mice, suggesting that ATF3 acts as a mammary oncogene. The resulting tumors aberrantly express cytokeratins that are characteristic of several mammary and epidermal lineages and are phenotypically similar to tumors produced in other transgenic models that upregulate Wnt/β-catenin signaling in the mammary gland. A subset of human breast tumors expresses high levels of ATF3, suggesting that ATF3 may play an oncogenic role in human breast tumorigenesis. A better understanding of the molecular mechanisms by which ATF3 overexpression initiates mammary tumorigenesis in the transgenic mouse model may provide new biomarkers and/or therapeutic targets for a subset of human breast tumors.

## Abbreviations

CK: cytokeratin; ERα: estrogen receptor alpha; IDC: invasive ductal carcinoma; IHC: immunohistochemistry; DCIS: ductal carcinoma *in situ*.

## Competing interests

The authors declare that they have no competing interests.

## Authors' contributions

AW developed the BK5.ATF3 transgenic mouse model. SA and LY characterized the phenotypes. KK developed the fluorescence IHC technique. MJM participated in the histopathological analysis of murine mammary lesions. AS provided human breast tumor samples and contributed to the analysis of ATF3 staining patterns in the tumors. HDT provided biostatistical support. CMA contributed to study design, data interpretation and histopathological analysis. MCM conceived and directed the project and prepared the manuscript. All authors read and approved the final draft of the manuscript.

## Pre-publication history

The pre-publication history for this paper can be accessed here:


